# Population Health Surveillance Using Mobile Phone Surveys in Low- and Middle-Income Countries: Methodology and Sample Representativeness of a Cross-sectional Survey of Live Poultry Exposure in Bangladesh

**DOI:** 10.2196/29020

**Published:** 2021-11-12

**Authors:** Isha Berry, Punam Mangtani, Mahbubur Rahman, Iqbal Ansary Khan, Sudipta Sarkar, Tanzila Naureen, Amy L Greer, Shaun K Morris, David N Fisman, Meerjady Sabrina Flora

**Affiliations:** 1 Dalla Lana School of Public Health University of Toronto Toronto, ON Canada; 2 London School of Hygiene and Tropical Medicine London United Kingdom; 3 Institute of Epidemiology, Disease Control and Research Dhaka Bangladesh; 4 Department of Population Medicine University of Guelph Guelph, ON Canada; 5 Division of Infectious Disease and Center for Global Child Health The Hospital for Sick Children Toronto, ON Canada

**Keywords:** mobile telephone survey, health surveillance, survey methodology, Bangladesh

## Abstract

**Background:**

Population-based health surveys are typically conducted using face-to-face household interviews in low- and middle-income countries (LMICs). However, telephone-based surveys are cheaper, faster, and can provide greater access to hard-to-reach or remote populations. The rapid growth in mobile phone ownership in LMICs provides a unique opportunity to implement novel data collection methods for population health surveys.

**Objective:**

This study aims to describe the development and population representativeness of a mobile phone survey measuring live poultry exposure in urban Bangladesh.

**Methods:**

A population-based, cross-sectional, mobile phone survey was conducted between September and November 2019 in North and South Dhaka City Corporations (DCC), Bangladesh, to measure live poultry exposure using a stratified probability sampling design. Data were collected using a computer-assisted telephone interview platform. The call operational data were summarized, and the participant data were weighted by age, sex, and education to the 2011 census. The demographic distribution of the weighted sample was compared with external sources to assess population representativeness.

**Results:**

A total of 5486 unique mobile phone numbers were dialed, with 1047 respondents completing the survey. The survey had an overall response rate of 52.2% (1047/2006) and a co-operation rate of 89.0% (1047/1176). Initial results comparing the sociodemographic profile of the survey sample to the census population showed that mobile phone sampling slightly underrepresented older individuals and overrepresented those with higher secondary education. After weighting, the demographic profile of the sample population matched well with the latest DCC census population profile.

**Conclusions:**

Probability-based mobile phone survey sampling and data collection methods produced a population-representative sample with minimal adjustment in DCC, Bangladesh. Mobile phone–based surveys can offer an efficient, economic, and robust way to conduct surveillance for population health outcomes, which has important implications for improving population health surveillance in LMICs.

## Introduction

### Background

Representative population-based surveys are important for measuring health outcomes and behavioral risk factors at the national and subnational levels [1]. These surveys can be used to provide key population health estimates, and if conducted at regular intervals, health trends can be monitored over time [1-3]. In low- and middle-income countries (LMICs), population health surveys are typically conducted using face-to-face household interviews due to methodological challenges, including a lack of representative individual sampling frames [4]. However, because of their high implementation costs and the time required, representative household surveys tend to be conducted only periodically [5]. In contrast, higher income countries (HICs) have developed and used annual telephone-based surveys, such as those for monitoring behavioral risk factor trends [6,7].

Telephone-based surveys are cheaper, faster, and can provide greater access to hard-to-reach or remote populations, particularly in the era of COVID-19, and hence are an appealing method for supplementing or replacing in-person household surveys in LMICs [8,9]. While sampling frames for probability-based telephone surveys have traditionally been limited to landlines in HICs, the growth of mobile phone ownership and the increasing number of mobile phone–only households has led to the development of dual-frame sampling designs [10,11]. However, in LMICs, the growth in mobile phone subscriptions has been exponential, with 22.9 subscriptions per 100 people in 2005 to 99.3 per 100 in 2020 [12]. This rapid increase has led to cellular networks leapfrogging landline infrastructure and mobile phones becoming the primary mode of communication [13].

High levels of phone ownership in LMICs provide a unique opportunity to implement novel data collection methods for population health surveys using mobile phones as a primary sampling unit [13]. However, there remain important methodological concerns regarding the use of mobile phone surveys in producing population-representative samples owing to sampling bias, coverage error, and low response rates [14]. For example, the sociodemographic profiles of mobile phone respondents have been shown to differ from those of face-to-face household survey respondents [15,16]. Recent systematic reviews identified only a few studies that were published using probability-based mobile phone survey methods in LMICs and reported a lack of consensus on the best implementation approaches and analytic methods to overcome methodological challenges in these populations [17,18].

In Bangladesh, where the mobile phone penetration rate is over 87% [19], phone-based surveys have been increasingly used for behavioral risk factor surveillance [20]. In urban areas, where the mobile phone penetration rate is even higher [21], these surveys have the potential to be especially useful for measuring population health outcomes. However, the population representativeness of these surveys has not been systematically evaluated and analytic methods such as poststratification adjustments have not been applied [20]. Therefore, the potential impact of sampling bias and coverage error on study findings and population estimates remains unknown.

### Objectives

This study aims to address these critical methodological gaps to support the use of probability-based mobile phone survey methods for routine population health surveillance in LMICs. Here we describe the development of a mobile phone survey for measuring live poultry exposure in urban Bangladesh. Human-animal contact is a significant risk factor for the emergence of novel infectious diseases [22] and is, therefore, a key measure to capture in behavioral risk factor surveillance. Specifically, we provide an in-depth discussion of the methods covering sample design, questionnaire development, data collection, and poststratification analytic methods, as well as call outcome results, including response rates and population representativeness.

## Methods

### Study Design and Sampling

We conducted a population-based cross-sectional mobile phone survey between September and November 2019 to recruit a representative sample of adult males and females in North and South Dhaka City Corporations (collectively known as Dhaka City Corporation; DCC), in Dhaka, the capital of Bangladesh. The sampling frame was a list of mobile phone numbers from each of Bangladesh’s 4 mobile phone operators (ie, Grameenphone, Robi Axia, Banglalink, and Teletalk). We restricted the phone numbers to those active in DCC, or if they could not be restricted to DCC, to those active in Dhaka district. Over 75% of the population of Dhaka district resides in DCC [23]. The phone numbers were provided by each mobile phone operator with permission from the Bangladesh Telecommunication Regulatory Commission.

We used a single-stage stratified probability sampling design to select participants. Before selection, the phone numbers were stratified by mobile operator and sampled in accordance with each operator’s proportionate market share to maximize the precision of the sample and to ensure a representative distribution (Multimedia Appendix 1, Table S1) [24]. Within each operator list, simple random sampling was used to select phone numbers. At the time of contact, we screened each selected mobile phone respondent for eligibility, and we recruited an equal number of male and female respondents to allow for robust sex-specific analyses. Individuals were eligible for inclusion if they were at least 18 years of age, were current DCC residents, and had been residing in DCC for the past 1 year.

### Questionnaire Development

The questionnaire was based on previous poultry exposure surveys conducted in urban China [25-28] but modified to the Bangladeshi context through discussions with a 12-member advisory panel consisting of local experts in survey design, mobile phone surveys, and infectious diseases. Using a structured approach, the panel reviewed each survey question to assess the face and content validity of the items, as well as to identify areas for potential adaptation or modification and item reduction or addition. We conducted 2 rounds of review, and any items that did not achieve group consensus (defined as 60% agreement) were modified and re-examined until consensus was reached. Key revisions in this step centered on prioritizing and selecting items that were deemed feasible and reliable to ask participants during a phone interview. The questionnaire was translated into Bangla and independently reviewed by 2 native speakers familiar with the content to ensure comprehension and clarity; any translation disagreements were reviewed and further updated by the study team.

The final survey instrument comprised 5 sections and captured information on individual and household exposure to live poultry when purchasing at live bird markets (LBMs) and preparing food, prevention practices, influenza-like illness, and sociodemographics. LBMs were defined as a collection of stalls or vendors where the public could purchase live chickens, ducks, geese, or any by-products of these in an unprocessed form [29]. Specifically, the questions covered the following topics: frequency of LBM visits and the associated behaviors in markets, poultry processing practices during food preparation, uptake and adherence to hygiene practices, use of personal protective equipment (ie, gloves, facemask, and apron) during and after poultry exposure, self-reported influenza-like illness using a standard case definition [30], and household and individual-level sociodemographics. To minimize respondent burden when obtaining detailed information, where appropriate, the survey used a significant amount of branching logic. The questionnaire was thoroughly reviewed, and modifications were made as needed based on feedback from a pretesting phase (n=7) and a small-scale pilot (n=41). The final updated survey took approximately 10 to 15 minutes to complete.

### Data Collection and Calling Procedure

We programmed both English and Bangla versions of the questionnaire into a customized computer-assisted telephone interview (CATI) platform developed by the Institute of Epidemiology, Disease Control and Research (IEDCR) in Dhaka, Bangladesh. This platform managed both the sampling and data collection processes, including complex form structure, automated repeat call attempts and interview rescheduling, automated strata monitoring of key variables (ie, mobile phone operator and sex of respondent) across interviewers, and pairing with a mobile phone app to facilitate automated dialing of each selected phone number. A team of 4 female data collectors was recruited to conduct the phone interviews, and data were entered into the CATI platform in real time. The data collectors received 4 days of training on the survey methods and questionnaire topics before the start of the pilot and data collection phases.

We conducted the survey between September and November 2019. In advance, we placed a Bangla-language newspaper advertisement in DCC’s 2 most circulated newspapers to inform the public that they might receive a call from IEDCR regarding a health survey, that the phone numbers were randomly selected with the permission of the Bangladesh Telecommunication Regulatory Commission, and that participation was important for improving population health. Phone calls were made every day of the week between 8 AM and 8 PM (local time), except on Friday afternoons because of local religious observances, to limit the potential sampling bias that could result from recruiting only during weekdays and working hours.

Our team attempted calling each phone number up to 4 times to establish contact and conduct an interview with the respondent. Each unanswered call was automatically rescheduled for a different time of the day on a different day of the week over the following 7-day period. If the respondent was not reached after the maximum number of 4 call attempts, with at least one daytime and one evening call attempt, the phone number was classified as *no contact* and discontinued. At the first successful contact, we explained to the respondent the purpose of the study, the survey length, that participation was voluntary, and that all the information they provided would be kept confidential. Eligibility was confirmed and consent for survey participation was obtained at the time of the interview. When respondents were unable to complete the interview at the time of recruitment, we rescheduled the phone interview to a convenient time within the next 7 days. Once an interview was completed, or if a respondent declined, refused, or was ineligible, we discontinued the phone number from the call bank. In line with the IEDCR phone-based disease surveillance practices, no compensation was provided for participation. An overview of the recruitment process is provided in Multimedia Appendix 1, Figure S1.

### Sample Size

A total of 1040 complete interviews (520 males and 520 females) were required to detect an 8% to 9% difference (65% vs 56% [26]) in live poultry exposure between strata, with 95% confidence and 80% power. The reason for explicitly stratifying by sex was to have sufficient statistical power to permit detailed exploration and identify notable differences in high-risk behaviors between males and females. This was important for ensuring appropriate and targeted risk-based implementation strategies.

### Ethics Approval

This study received ethical approval from the committees of each of the participating research institutions: University of Toronto (protocol no. 37657), the Institute of Epidemiology, Disease Control and Research (IRB/2019/11), and the London School of Hygiene and Tropical Medicine (ref 17661). All the participants provided oral informed consent via phone.

### Data Analysis: Response, Weighting, and Representativeness

The operational data for each phone number dialed and the corresponding details for call outcome status were summarized. We calculated the overall and mobile operator–specific response rates according to the American Association for Public Opinion Research (AAPOR) Response Rate-3 definition, which included those who were eligible and those estimated to be eligible in the denominator [31]. The number of persons estimated to be eligible was derived by assuming that the proportion of eligible individuals among those contacted was the same as for those who could not be contacted or who declined before their eligibility was determined.

The demographic data for completed interviews were summarized, and the sample distributions were compared with the Dhaka City Corporation demographic profile of the 2011 census [23]. To adjust for nonresponse and disproportionate stratified sampling by sex (ie, oversampling of females compared with the reference population), poststratification weights were calculated by age, sex, and education to align with the 2011 census. Weights were calculated using the census population fraction_ijk_/study population fraction_ijk_, where i was the age category, j was sex, and k was the highest completed education level. No extreme values were identified upon inspection. Participants with an invalid response to the weighting variables (ie, age, sex, and education) could not be assigned a weight and were, therefore, not included in the weighted analyses (n=16). The demographic distribution of the weighted data was summarized and compared with external data sources to assess the representativeness of the sample population for other key demographic variables, including marital status and region. All analyses were conducted in Stata 16.1 (StataCorp).

## Results

### Call Outcomes and Response Rates

**Figure 1:**
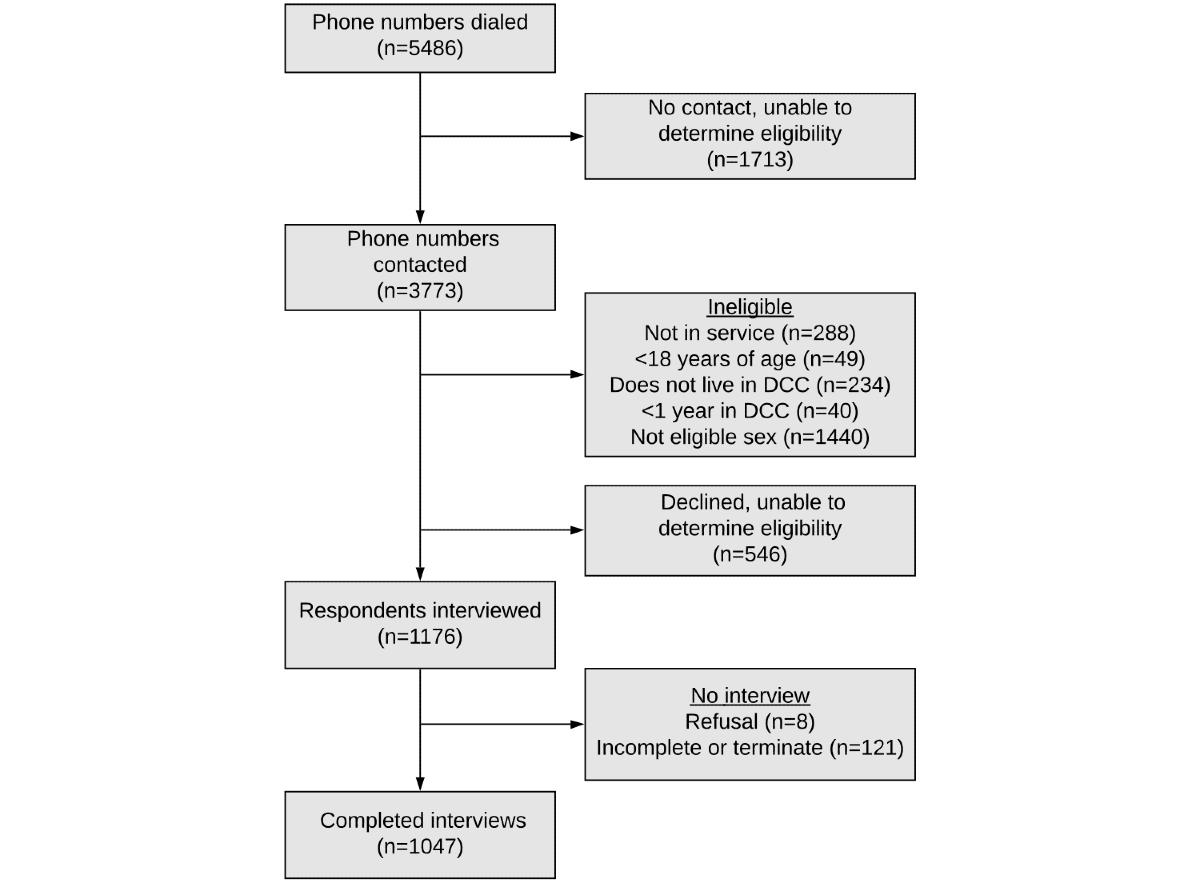
Profile of participant recruitment and call outcome classification for the live poultry exposure mobile phone survey, Dhaka City Corporation, Bangladesh. DCC: Dhaka City Corporation.

Between September and November 2019, 5486 unique phone numbers were dialed. An overview of participant recruitment and outcome classifications is shown in Figure 1. Of the 5486 phone numbers dialed, 2051 (37.4%) were screened and determined to be ineligible. This included 288 (5.2%) phone numbers that were not in service, 49 (0.9%) respondents who were not in the eligible age range, 234 (4.3%) respondents who were not living in DCC, and 40 (0.7%) who had been living in DCC for less than 1 year. In addition, 1440 (26.2%) respondents were excluded because their sex-specific strata were complete; no information was obtained from 2259 (41.2%) phone numbers, including 1713 (31.2%) with no contact and 546 (10.0%) where the respondent declined to participate. Assuming that the mobile operator-weighted proportion of eligible individuals among those contacted and screened for eligibility was the same as for those who could not be contacted or who declined, 830 (36.7%) of these 2259 phone numbers were estimated to be eligible. Interviews were completed with 1047 (52.2%) respondents out of the 2006 eligible (known and estimated) phone numbers. The overall cooperation rate was 89.0%, based on the number of known eligible respondents contacted. Table 1 presents the call outcomes and response rates overall and by mobile phone operator.

**Table 1:** Call outcome classification and response rates overall and by mobile phone operator for the live poultry exposure mobile phone survey, Dhaka City Corporation, Bangladesh.

Call outcome status					All, n (%)		Grameenphone, n (%)		Robi Axia, n (%)		Banglalink, n (%)		Teletalk, n (%)
**Call outcomes**													
	Phone numbers dialed				5486 (100)		1901 (100)		2100 (100)		1346 (100)		139 (100)
	**Total ineligible**				2051 (37.4)		697 (36.7)		932 (44.4)		367 (27.3)		55 (39.6)
		Not in service			288 (5.2)		42 (2.2)		115 (5.5)		126 (9.4)		5 (3.6)
		<18 years of age			49 (0.9)		15 (0.8)		13 (0.6)		18 (1.3)		3 (2.2)
		Does not live in DCC^a^			234 (4.3)		89 (4.7)		31 (1.5)		113 (8.4)		1 (0.7)
		Less than 1 year in DCC			40 (0.7)		20 (1.1)		5 (0.2)		13 (1.0)		2 (1.4)
		Not eligible sex (strata complete)			1440 (26.2)		531 (27.9)		768 (36.6)		97 (7.2)		44 (31.7)
	**Total unknown eligibility**				2259 (41.2)		663 (34.9)		817 (38.9)		723 (53.7)		56 (40.3)
		No contact			1713 (31.2)		441 (23.2)		643 (30.6)		590 (43.8)		39 (28.1)
		Declined (no eligibility screening)			546 (10.0)		222 (11.7)		174 (8.3)		133 (9.9)		17 (12.2)
**Response rates**													
	**Total eligible (known + estimated)**				2006 (100)		831 (100)		575 (100)		553 (100)		47 (100)
		**No interview**											
			Refusal	8 (0.4)		0 (0)		2 (0.3)		6 (1.1)		0 (0)	
			Incomplete or terminated	121 (6.0)		54 (6.5)		39 (6.8)		26 (4.7)		2 (4.3)	
		No information (estimated eligible)^b^			830 (41.4)		290 (34.9)		224 (38.9)		297 (53.7)		19 (40.3)
	Complete interviews (response rate)				1047 (52.2)		487 (58.6)		310 (54.0)		224 (40.5)		26 (55.4)

^a^DCC: Dhaka City Corporation.

^b^Estimated eligible number calculated using American Association for Public Opinion Research (AAPOR) guidelines [31], assuming that the proportion of eligible individuals among those who were contacted and screened is the same as for those who were unable to be contacted or declined.

### Sample Characteristics and Representativeness

Compared with the DCC demographic profile from the 2011 census, the unweighted mobile phone survey sample overrepresented males aged 25 to 34 years as well as males and females with higher secondary education, while it underrepresented males and females aged 55 to 74 years and those with primary or lower than primary education. Given the stratified sampling design aimed at achieving equal representation of males and females, the overall unweighted sample overrepresented females and underrepresented males. After poststratification weighting on these key variables, the sample closely matched the population in terms of age, sex, and education (Table 2).

The weighted survey sample is representative of other demographic factors that were not used in the construction of the weights (Table 3). The overall sample shows a close match to the 2011 census figures for DCC by region, with slight discrepancies within the sex-specific strata. In terms of marital status, the survey slightly underrepresented single males and married females.

**Table 2:** Comparison of unweighted and weighted survey sample with 2011 census population benchmarks for the live poultry exposure mobile phone survey, Dhaka City Corporation, Bangladesh.

Characteristics		Unweighted sample				Weighted sample^a^				Census benchmarks^b^		
		Male (%)	Female (%)	All (%)	Male (%)		Female (%)	All (%)	Male (%)		Female (%)	All (%)
**Age group (years)**												
	18-24	25.4	26.4	25.9	25.4		29.3	27.1	25.4		29.3	27.1
	25-34	40.6	31.0	35.8	32.7		32.3	32.5	32.7		32.3	32.5
	35-44	19.0	23.8	21.4	20.9		19.7	20.4	20.9		19.7	20.4
	45-54	9.2	12.8	11.0	12.1		11.0	11.6	12.1		11.0	11.6
	55-74	5.8	5.9	5.9	8.9		7.7	8.4	8.9		7.7	8.4
**Sex**												
	Male	N/A^c^	N/A	50.0	N/A		N/A	57.5	N/A		N/A	57.5
	Female	N/A	N/A	50.0	N/A		N/A	42.5	N/A		N/A	42.5
**Education (highest completed)**												
	<Primary^d^	10.0	10.1	10.1	20.8		28.1	23.9	20.8		28.1	23.9
	Primary	28.8	24.6	26.7	30.9		31.4	31.1	30.9		31.4	31.1
	Secondary^e^	11.2	14.7	13.0	12.5		13.3	12.8	12.5		13.3	12.8
	≥Higher secondary^f^	50.0	50.7	50.3	35.8		27.2	32.2	35.8		27.2	32.2

^a^Sample weighted by age, sex, and education to the Dhaka City Corporation demographic profile of the 2011 census.

^b^Census data for the 2011 Dhaka City Corporation demographic profile [23].

^c^N/A: not applicable.

^d^Primary indicates year 5 of school.

^e^Secondary indicates year 10 of school.

^f^Higher secondary indicates year 12 of school.

**Table 3:** Sample distribution compared with 2011 census population benchmarks for the live poultry exposure phone survey, Dhaka City Corporation, Bangladesh.

Characteristic			Weighted sample^a^						Census benchmarks^b^				
			Male (%)		Female (%)		All (%)		Male (%)		Female (%)		All (%)
**Marital status**													
	Single, never married	27.0		12.1		20.6		29.7		11.6		22.1	
	Married	71.2		78.7		74.4		69.8		80.9		74.5	
	Other^c^	1.8		9.2		4.9		0.6		7.4		3.4	
**Region**													
	DCC^d^ North	50.3		60.5		54.6		53.3		55.6		54.3	
	DCC South	49.7		39.5		45.4		46.7		44.4		45.7	

^a^Sample weighted by age, sex, and education to the DCC demographic profile of the 2011 census.

^b^Census data for the 2011 DCC population, aged 20 to 74 years for marital status and all ages for region.

^c^Other includes widows or widowers and divorced individuals.

^d^DCC: Dhaka City Corporation.

## Discussion

### Principal Findings

This study provides empirical evidence that probability-based mobile phone surveys can achieve population-representative samples with relatively high response rates (1047/2006, 52.2%) in urban Bangladesh. Although the unweighted sociodemographic profile of the survey showed that mobile phone sampling slightly underrepresented older individuals and overrepresented those with higher secondary education compared with the census population, poststratification weighting on a key set of demographic variables (age, sex, and education) was sufficient to correct for these differences. Therefore, these findings support the use of mobile phone–based survey sampling and data collection methods for producing population-representative samples with minimal adjustment in urban areas that have high mobile phone penetration. This has important implications for improving population surveillance in LMICs.

A response rate of approximately 50% is lower than that of previous phone-based surveys conducted in Bangladesh [20,32] but is in line with the response rates achieved in surveys conducted through similar methods in other LMICs and is in fact higher than those typically achieved in HICs [16,33]. Several factors might have contributed to this lower rate compared with previous work, including changes in the methods of calculating response rates over time to provide more conservative estimates and general declines in response rates of population health studies over the past 30 years [31,34,35]. The use of airtime incentives has been found to improve response rates in interactive voice response surveys in Bangladesh [36] and could also be explored as a method to increase the rates in CATIs. The response rates were generally similar across mobile phone operators, except for Banglalink, which was considerably lower. This could be owing to differences in the geographic sampling frames between each operator, with Banglalink not restricted to DCC and instead sampling from phone numbers listed in Dhaka district. Overall, this supports the use of stratified sampling designs by mobile phone operators to appropriately capture subpopulation heterogeneity when conducting population-based surveys [37].

The unweighted demographic profile of our sample differed most from the census population by educational attainment. Overrepresentation of respondents with higher education is consistent across survey research methods, including those conducted in LMICs and HICs [14,16,38]. The impact of these differences on population-level estimates would be greatest in surveys where education is strongly associated with the outcome of interest [39]. However, the magnitude of this impact becomes negligible once weighted to the distribution of the reference population [15,39]. Previous research in LMICs has found that minimal adjustment of demographic factors is sufficient to reduce nonresponse and coverage error when conducting robust probability-based sampling [15]. In addition, comparisons with the census population showed that our weighted survey achieved a good representation of other characteristics, including region and marital status. The remaining differences between the census and the survey population distributions could be because our survey sample included only participants aged 18 to 74 years, whereas the published census figures include all ages for region and only ages 20 to 74 years for marital status. By conducting sex-stratified sampling, our study was sufficiently powered to explore sex-based subgroup analyses without generating extreme survey weights. Researchers intending to conduct similar mobile phone–based surveys with poststratification weights should determine subgroup analyses a priori to ensure that adequate stratified sampling is implemented.

### Limitations

Although we demonstrated that population-based probability sampling using mobile phones can produce representative samples with minimal adjustment, this method had some limitations, as evidenced in this study. First, although comparisons to the census population can be used to evaluate the representativeness of the sample population, it does not preclude the potential for sampling bias due to coverage error. For instance, individuals who do not have mobile phones are likely different from those who do, based on factors such as socioeconomic status [40,41]. However, in DCC the mobile penetration rate was very high at over 87% [19], which suggests that impacts on population estimates due to coverage error were likely minimal in this urban area. Further work could examine this question in populations with lower mobile phone coverage or in nonurban settings where mobile phones may be shared within and between households, which would necessitate additional strategies to achieve a representative sample (eg, to randomly select an individual in a household using the same phone). The opposite is also of concern—potential bias introduced because of some individuals in the population having multiple mobile phone numbers. Recent studies quantifying this effect on the probability of selection have found that although the theoretical probability of inclusion for those with multiple phone numbers is greater than those with only one number, the likelihood of contacting any individual is extremely small in practice [13]. Although out of the scope of our analysis, this could be examined in future work by capturing information on mobile phone ownership and applying selection weights. The collection of additional household-based asset data to enable weighting by socioeconomic indicators, such as wealth index, could also be examined. In this survey, we only included education, which, while strongly correlated with relative wealth quintile, does not explicitly capture wealth [42]. However, examining the impact of increasing the survey length to capture household-based assets data on response rates is warranted. Finally, poststratification survey weighting should be conducted using recent reference population estimates. However, for DCC, the most recent census available is from 2011 [23]; therefore, any significant changes in the underlying population distribution during this time would not be reflected in the weights and could impact weighted population estimates.

### Conclusions

In many LMICs, such as Bangladesh, the coverage of mobile phones is very high and includes a range of population subgroups. Mobile phone–based surveys can, therefore, offer an efficient, economic, and robust way to conduct surveillance for population health outcomes. We found that a mobile phone survey using a stratified probability sampling design produced a population-representative sample with minimal adjustment in urban Bangladesh. These results have important implications for improving population health surveillance methods in LMICs.

## Appendix

Multimedia Appendix 1 Additional information on mobile phone operators’ proportionate market share and phone calling procedures.

## Abbreviations

CATI: computer-assisted phone interview
DCC: Dhaka City Corporation
HIC: higher income country
IEDCR: Institute of Epidemiology, Disease Control and Research
LBM: live bird market
LMIC: low- and middle-income country
