# Supplementary material for: Population Health Surveillance Using Mobile Phone Surveys in Low- and Middle-Income Countries: Methodology and Sample Representativeness of a Cross-sectional Survey of Live Poultry Exposure in Bangladesh
Source: JMIR Public Health Surveill. 2021 Nov 12;7(11):e29020. doi: 10.2196/29020 (PMC8663489; doi:10.2196/29020)
Supplement: Multimedia Appendix 1 [file publichealth_v7i11e29020_app1.doc]

**Title:** Population Health Surveillance Using Mobile Phone Surveys in Low- and Middle-Income Countries: Methodology and Sample Representativeness of a Cross-Sectional Survey of Live Poultry Exposure in Bangladesh

**Authors & Affiliations:** Isha Berry^1^, Punam Mangtani^2^, Mahbubur Rahman^3^, Iqbal Ansary Khan^3^, Sudipta Sarkar^3^, Tanzila Naureen^3^, Amy L. Greer^1,4^, Shaun K. Morris^1,5^, David N. Fisman^1^, Meerjady Sabrina Flora^3^

^1^Dalla Lana School of Public Health, University of Toronto, Toronto, Canada.

^2^London School of Hygiene and Tropical Medicine, London, United Kingdom.

^3^Institute of Epidemiology, Disease Control and Research, Dhaka, Bangladesh.

^4^Ontario Veterinary College, University of Guelph, Guelph, Canada.

^5^Division of Infectious Disease and Center for Global Child Health, The Hospital for Sick Children Toronto, Canada.

**Correspondence:** Isha Berry, Dalla Lana School of Public Health, University of Toronto, Toronto, Ontario, Canada; isha.berry@mail.utoronto.ca

**Supplementary Table S1.** Proportionate market share of each mobile phone operator and corresponding breakdown of sampling frame for the live poultry exposure mobile phone survey, Dhaka City Corporation, Bangladesh

| **Mobile Phone Operator** | **Subscribers (millions)^a^** | **Market Share (%)** | **Target Interviews** | **Sampling Frame Coverage Area** |
| --- | --- | --- | --- | --- |
| Total | 161.772 | 100.0 | 1040 |  |
| Grameenphone Ltd. | 75.330 | 46.6 | 485 | DCC |
| Robi Axiata Ltd. | 47.939 | 29.6 | 310 | DCC |
| Banglalink Digital Communications Ltd. | 34.667 | 21.4 | 224 | Dhaka district |
| Teletalk Bangladesh Ltd. | 3.836 | 2.4 | 26 | Dhaka district |
| **Note**: Ltd., limited; DCC, Dhaka City Corporation  ^a^Total number of mobile phone subscribers defined as biometrically verified phone number with any voice, data, messaging activity at least once in the preceding 90 days, as of June 2019 [24]. | | | | |

**Supplementary Figure S1.** Mobile phone sampling calling procedure and call outcome categories for the live poultry exposure mobile phone survey, Dhaka City Corporation, Bangladesh
